# Swindling in Life Assurance

**Published:** 1900-07-21

**Authors:** 


### SWINDLING IN LIFE ASSURANCE.

Those engaged in examining persons for life assurance have to exercise the greatest care and judgment in many directions, but above all they have to be constantly on the *qui vive* for swindling. Dr. Vivian Poore[1] describes how one may be taken in with regard to the examination of the urine, for it is unfortunately the fact that people are quite ready to set traps for one in this matter. "I remember a man," he says, "coming in to insure his life. He was very smart, and had on a big fur coat. I said to him, 'Will you pass me a little urine into this vessel?' I sat at a table writing, and I noticed that the sound of his passing urine was very odd; it was a succession of pops, like the sound made by water coming out of a narrow-necked bottle. I looked up from where I was writing, and I could not see what he was doing because of the big coat. But as he finished I saw his hand go into his side pocket. You have always to be looking out for fraud, and it crossed my mind that the urine was not his own. But, on the other hand, it would be very awkward to make a mistake; it would be very awkward to wrongly accuse a man of fraud of that kind. I confess I was in very considerable doubt as to what to do until he put the glass beaker into my hand. Directly I got it into my hand I knew what to do. I said, 'We cannot take you; you must be exceedingly ill; this urine is as thick as mustard and as cold as ice.' We found he had been trying to deceive the people at another office. He had brought somebody else's urine in a bottle. There was no albumen in the urine he gave me, but he had been rejected elsewhere for albuminuria, and some of his urine obtained subsequently was found to be albuminous." This is a good example of the tricks by which "respectable" members of the community may at one time or another endeavour to come round the examiner for life assurance.

[1] Clinical Jour., July 4.

